# Changes in nomenclature, virulence factors, and antifungal resistance of the genus *Candida*

**DOI:** 10.3389/ffunb.2025.1677892

**Published:** 2025-10-15

**Authors:** Maria Alyce Albuquerque Fernandes, Francisca Lidiane Linhares de Aguiar, Maria Gleiciane Soares Coutinho, Erika Helena Salles de Brito, Camila Gomes Virginio Coelho, Raquel Oliveira dos Santos Fontenelle

**Affiliations:** ^1^ Postgraduate Program in Health Sciences, Federal University of Ceará, Sobral, Ceará, Brazil; ^2^ Microbiology Laboratory (LABMIC), Vale do Acaraú State University, Sobral, Ceará, Brazil; ^3^ Institute of Health Sciences, University for International Integration of Afro-Brazilian Lusophony, Redenção, Ceará, Brazil; ^4^ Faculty of Medicine, Federal University of Ceará, Sobral, Ceará, Brazil

**Keywords:** *Candida* spp., taxonomic changes, virulence, resistance mechanisms, antifungals

## Abstract

Some *Candida* species of clinical interest have undergone recent nomenclature changes. These yeasts have a high capacity to adhere to and infect host tissues, driven by their virulence factors, as well as by the incidence of antifungal resistance. This review aimed to analyze the taxonomic changes of the main species of clinical interest within the *Candida* genus, considering the clinical implications of their virulence factors and the main mechanisms of antifungal resistance. The research results allowed us to understand that the updated nomenclature of *Candida* species is essential to maintain the criteria that define a genus, organizing the species according to their phylogenetic and evolutionary characteristics. Understanding the virulence factors and resistance mechanisms of the different species of clinical interest helps us understand how infections are initiated and established, as well as how these same species behave to neutralize the action of antifungals. Therefore, integrating knowledge of taxonomy, virulence, and resistance profiles is crucial for effective strategies to control and treat fungal infections.

## Introduction

According to taxonomy, fungi are classified based on their phylogenetic characteristics, grouping organisms that share similar evolutionary traits. However, due to the complexity of fungi, taxonomic classification is subject to modifications over time to meet the criteria that define a genus. Some of the main *Candida* species have undergone changes in nomenclature, being renamed, subdivided within species, and reallocated into new clades (Kidd et al, 2023; Takashima and Sugita, 2022).

Yeasts of the genus *Candida* are unicellular microorganisms with spherical or oval shapes that live in the environment or are commonly part of the human microbiota, where they can be opportunistic pathogens. Some species can alter their morphology to a filamentous form, which is essential for adhesion and invasion of host cells (Lim et al., 2021; Pappas et al., 2018). This ability to perform morphological adaptations is one of the virulence factors presented by the genus, which favors the survival of the species in specific environments while facilitating superficial and invasive infections (Pappas et al., 2018).

*Candida* spp. are gaining more attention due to their ability to infect hosts and the high incidence of hospital-acquired invasive infections. They are recognized as a major cause of morbidity and mortality, stemming from their virulence factors that contribute to decreasing or nullifying the effect of antifungals (Pappas et al., 2016, Pappas et al, 2018). According to Baptista et al. (2020), approximately 80% of fungal infections reported in tertiary hospitals are attributed to yeast infections of the *Candida* genus.

Virulence factors not only increase the pathogenicity of *Candida* spp. but may also contribute to the antifungal resistance mechanisms developed by yeasts, allowing them to evade drug action. Antifungal resistance can be intrinsic or acquired, with reports of *Candida* spp. resistance to standard antifungals recurring (Czajka et al., 2023; Pappas et al., 2018; Thanyasrisung et al., 2023). Among the main antifungal resistance mechanisms presented by the *Candida* genus are genetic and enzymatic modifications, activation of efflux pumps, and those presented by biofilms (Miron-Ocampo et al., 2023; Nett and Andes, 2020). In view of the above, this review work aimed to analyze the taxonomic changes of the main species of clinical interest of the *Candida* genus, considering the clinical implications of their virulence factors and the main mechanisms of resistance to antifungals.

## Changes in *Candida* spp. nomenclature

According to Kidd et al. (2023), over the past decade, fungal nomenclature has undergone significant transformations. This is due to advances in molecular technologies in taxonomy, diagnostics, and epidemiology, all aimed at meeting the criteria for defining a genus, such as monophyly, the range of species within a genus, and shared evolutionary and phylogenetic characteristics. Effective January 1, 2013, the practice of using different names for the teleomorph (sexual) and anamorph (asexual) states of fungi was prohibited. Therefore, mycologists must choose a single name from among the many that often already exist for the same species (Borman and Johnson, 2021).

The group of fungi that has undergone the most recent reclassifications and is causing the greatest concern among physicians and medical laboratories are the species of the genus *Candida* (Kidd et al., 2023). The genus *Candida* belongs to the phylum Ascomycota, class Saccharomycetes, and family Debaryomycetaceae. Multigene phylogenetic analyses suggest that the species of this genus can be grouped into more than 10 distinct clades, with occasional name changes and intraspecies subdivisions (Takashima and Sugita, 2022).

Among the species of clinical interest that have undergone reclassification are *Candida glabrata*, *Candida krusei*, *Candida guilliermondii*, and *Candida lusitaniae*, which are now named *Nakaseomyces glabrata* (*Nakaseomyces*), *Pichia kudriavzevii* (*Pichiaceae*), *Meyerozyma guilliermondii* (*Debaryomycetaceae*), and *Clavispora lusitaniae* (*Metschnikowiaceae*), respectively (Kidd et al., 2023; Takashima and Sugita, 2022). *Candida parapsilosis* has also undergone recent changes, remaining in the same clade, *Lodderomyces*, and has been subdivided into three: *C. parapsilosis*, *C. orthopsilosis*, and *C. metapsilosis*, also known as the *Candida parapsilosis* complex. The three species were organized as a single species for a long time (Takashima and Sugita, 2022; Govrins and Lass-Flörl, 2024).

The species *C. albicans*, *C. parapsilosis*, and *C. tropicalis*, which belong to the *Lodderomyces* clade, one of the largest clades with proven monophyly, retained the name *Candida* (Kidd et al., 2023; Stavrou et al., 2019; Takashima and Sugita, 2022). *Candida auris*, a multiresistant species, despite being part of the *Metschnikovi*a clade, retained the same name (Kidd et al., 2023). New changes were made, and *C. auris* is now part of *Candidozyma* with the new nomenclature of *Candidozyma auris* (Liu et al., 2024).

## Main virulence factors

### Adhesion

Adhesion to host cells represents a critical step in the establishment of *Candida* spp. infections and is considered one of the first and most important virulence factors of these opportunistic yeasts. This process is mediated by specialized proteins called adhesins, which are located on the fungal cell surface and facilitate attachment to biotic and abiotic substrates (Nobile and Johnson, 2015; Alim et al, 2018; Wall et al., 2019). An important family of adhesins are the *Als* (Agglutinin-like sequence) proteins, particularly *Als1*, *Als3*, and *Als5*, which have high binding affinity for extracellular matrix components such as fibronectin, laminin, and collagen, favoring adhesion to epithelial and endothelial cells. *Als* expression is regulated by environmental factors and the morphological state of the cell, being intensified during the transition to the filamentous form (Lombardi et al., 2019; Oh et al., 2021; Pokhrel et al., 2022; Bing et al., 2023).

*C. albicans* also contains adhesins of the *Hwp* (Hyphal wall protein) family, which are associated with the formation of true hyphae. The *Hwp1* protein binds to host cells, enabling the formation of covalent bonds between the fungus and epithelial surface proteins. This interaction is highly stable and confers adhesion that is resistant to fluid mechanics and the local immune response. It is primarily expressed under conditions that favor filamentous growth, such as the presence of serum and neutral pH, commonly found in host tissues during the infection phase (Nobile and Johnson, 2015; Pokhrel et al., 2022; Wooten et al., 2021).

*N. glabrata*, which does not form a filamentous structure, presents a distinct pathogenic profile. The adhesion process is mediated by a different family of adhesins, the *EPA* (Epithelial adhesins) (Valotteau et al., 2019). *EPA1*, *EPA6*, and *EPA7* are primarily responsible for adhesion to human epithelial cells and vascular endothelium. *EPA1*-mediated adhesion occurs through the recognition of specific glycans on the surface of host cells, functioning as a highly specific ligand (López-Fuentes et al., 2018; Yu et al, 2018; Hassan et al, 2021).

### Hydrolytic enzymes

The enzymes most frequently associated with the pathogenicity of *Candida* spp. are proteinases (SAPs), which act on albumin, the extracellular matrix, and host immunoglobulins. These enzymes are important in the process of adhesion to substrates, helping *Candida* species initiate the infectious process in host cells. They also have the unique characteristic of favoring the formation of pseudohyphae and hyphae, which are essential structures for tissue invasion (Czechowicz et al., 2022; Silva-Rocha et al., 2015).

Phospholipases, which break the ester bonds of phospholipids, facilitating yeast invasion. These enzymes are found on the surface of yeast and in germ tubes, playing a crucial role in the establishment of infection by degrading the phospholipid membrane of host cells. This degradation results in altered cellular characteristics, promoting greater adhesion of yeast to epithelial cells and medical devices, such as catheters and prostheses (Lim et al., 2021; Mroczyńska and Brillowska-Dąbrowska, 2021; Puello et al, 2023).

Lipase, which catalyzes the hydrolysis of triacylglycerols, and hemolysins are used by *Candida* species to degrade hemoglobin, promoting erythrocyte lysis, releasing iron, an essential nutrient for the growth and maintenance of fungal cells, and contributing to the survival of the pathogen in nutrient-limited environments (Lim et al., 2021; Mroczyńska and Brillowska-Dąbrowska, 2021; Nouraei et al., 2020).

### Polymorphism

Morphological change is a key process in the transition of most *Candida* spp. from commensal to pathogenic, where the filamentous form is essential for invasion into host cells and medical devices. Among the species that can change their morphology from yeast to pseudohyphae and true hyphae are *C. albicans* and *C. tropicalis*, but to a lesser extent, thus being polymorphic species (Czechowicz et al., 2022; Talapko et al., 2021; Wu et al., 2016). *C. albicans* has the ability to form structures called germ tubes, which are young true hyphae that form when in contact with host blood cells, providing greater adhesion to substrates (Jung et al., 2020; Trovato et al., 2020). The only other species known to also have the ability to form a germ tube is *C. dubliniensis* (Navarathna et al., 2016; Sampath et al., 2017).

*P. kudriavzevii, C. parapsilosis*, and *C. auris* can produce pseudohyphae and are considered dimorphic species (Czechowicz et al., 2022; Du et al., 2020; Pitarch et al., 2018). *N. glabrata* develops only in its yeast form (blastoconidia), which is also its pathogenic morphology (Czechowicz et al., 2022; Pitarch et al., 2018). The absence of morphological change directly influences its clinical behavior and survival strategies in the host. The absence of filamentous forms limits its direct invasive capacity in tissues compared to other *Candida* species. However, *N. glabrata* compensates for this limitation with increased adhesion capacity to surfaces and formation of more resistant biofilms, factors that make eradication of the infection difficult (López-Fuentes et al., 2018; Olson et al, 2018; Yu et al, 2018; Frías-De-León et al., 2021).

### Biofilm

Biofilms are highly organized biological communities where *Candida* spp. cells cluster together to form coordinated and functional structures. The cells can be mixed, composed of yeast, hyphae, and pseudohyphae, or solely yeast-like cells, such as the *N. glabrata* biofilm. They are immersed in a self-secreted extracellular matrix composed of proteins, carbohydrates, lipids, and DNA, establishing a complex three-dimensional structure that favors the entry of nutrients, the removal of waste, and the formation of microniches within the biofilm (Alim et al, 2018; Ghannoum et al., 2015; Silva et al., 2017; Wall et al., 2019).

These structures are regulated by a communication system called quorum sensing, where the cells of the forming biofilm communicate through lipid signals that control characteristics such as survival, pathogenicity factors, virulence, and behaviors in response to environmental conditions (Alim et al, 2018; Atriwal et al., 2021; Wall et al., 2019). Their formation is regulated by molecular processes in four distinct phases. Initially, yeast cells adhere to a surface, forming a base for the biofilm; over time, the cells proliferate and may develop filamentous structures, contributing to biofilm stability. During the maturation phase, the biofilm thickens due to the growth of the extracellular matrix, and its organization becomes three-dimensional. Eventually, dispersion occurs, in which cells detach from the biofilm and spread to other locations (Alim et al, 2018; Wall et al., 2019; Atriwal et al., 2021).

*Candida* species have a remarkable ability to form biofilms and are frequently found on hospital devices, dentures, prostheses, and especially catheters. On catheters, biofilms can develop intra and extraluminal adhesions (Alim et al, 2018; Atriwal et al., 2021; Silva et al., 2017; Wall et al., 2019). Biofilm formation on medical devices poses a significant clinical challenge, especially in hospital settings, conferring resistance to antifungals and hindering the immune system’s action, making infections persistent and difficult to treat (Thomaz et al., 2018; Jung et al., 2020; Melo et al., 2023).

Biofilm formation is one of the main reasons for antifungal treatment failure, as biofilm cells are protected from environmental stress and host defenses (Alim et al, 2018; Wall et al., 2019). Biofilms are difficult to treat due to their physical and genetic properties, presenting three main resistance mechanisms: the extracellular matrix that protects the cells, hindering the action of host defense cells and antifungal therapy; persister cells that acquire tolerance due to prolonged exposure to antifungals; and the activation of efflux pumps that occur during the primary stage of biofilm formation, adhesion (Nobile and Johnson, 2015; Silva et al., 2017; Atriwal et al., 2021; Kaur and Nobile, 2023).

## Main mechanisms of planktonic cell resistance

The three main classes of antifungals are azoles, polyenes, and echinocandins, which have distinct targets of action. Azoles inhibit the fungal enzyme lanosterol 14-α-demethylase, encoded by the cytochrome P450 gene *ERG11*, which is the enzyme involved in ergosterol synthesis (Chang et al., 2017; Salazar et al., 2020). Among polyenes, the most commonly used drug is Amphotericin B; its mechanism of action is fungal cell death through the formation of pores in ergosterol-containing membranes (Chang et al., 2017; Salazar et al., 2020). And echinocandins act by inhibiting β-1,3-glucan synthase, an enzyme complex that acts in the synthesis of the fungal cell wall (Chang et al., 2017).

The resistance mechanisms developed by *Candida* species to antifungal classes may occur due to mutations in the *ERG* genes, reducing the effectiveness of azoles, or by amino acid substitutions near the 14-α-demethylase binding site and by activation of efflux pumps (Campoy and Adrio, 2017; Houšť et al, 2020; Salazar et al., 2020). Resistance to polyenes is considered very rare (Chang et al., 2017; Salazar et al., 2020), but the mechanisms involved are alterations that result from mutations in the *ERG3* genes (Chang et al., 2017; Houšť et al, 2020; Salazar et al., 2020). The mechanisms of resistance to echinocandins are mutations in the *FKS1* and *FKS2* genes that result in amino acid substitutions in *Hs1* and *Hs2*, which are called “hotspots”, which are regions of the *FKS* genes that act in the synthesis of the fungal cell wall (Chang et al., 2017; Daneshnia et al., 2023).

## Discussion

According to Gabaldón et al (2016), *Candida* species are distributed throughout the Saccharomycotina phylogeny, present in most clades, and generally mixed with species from other genera, highlighting polyphyly, which differs from the criteria for genus definition. To adhere to taxonomic principles, new redefinitions are necessary so that species are organized within clades that suit their evolutionary and phylogenetic characteristics.

Adhesion mechanisms have important clinical implications that facilitate infection persistence. Their action in conjunction with hydrolytic enzymes contributes to evading the host’s immune response by modifying the local microenvironment (Talapko et al., 2021; Branco et al, 2023). Morphological transition is a key factor in the pathogenicity of most *Candida* species of clinical interest, associated with greater antifungal resistance, especially in biofilm infections. Therefore, they represent potential targets for new therapeutic strategies aimed at returning to the commensal stage and preventing biofilm formation.

Biofilm characteristics can vary according to the *Candida* species, presenting different responses to available therapies. In the study by Alves et al. (2023), the biofilm formed by isolates obtained from children had higher biomass and a matrix composition richer in polysaccharides than that isolated from adults. *P. kudriavzevii* was the most frequently isolated species in the children’s oral microbiota, and strains of this species also had higher biofilm biomass. The most frequently isolated species in adults was *C. albicans*, but it had a lower biofilm-forming capacity. The susceptibility of *Candida* spp. to antimicrobials in the biofilm of strains from the children’s group was directly associated with the amount of protein/polysaccharides, whereas no such relationship was observed in biofilms of strains from the adult group.

Fungal infections caused by planktonic cells intensify with biofilm formation, especially on medical devices, requiring differentiated therapeutic approaches. The activation and upregulation of efflux pumps in planktonic cells is triggered by the presence of antifungal drugs. In biofilm cells, this upregulation occurs naturally from the first hours of adhesion and persists throughout biofilm development, regardless of the presence of antifungal drugs (Nobile and Johnson, 2015; Kaur and Nobile, 2023). The distinct ways in which cells activate this mechanism help us understand the molecular processes involved in *Candida* spp. resistance due to efflux overexpression, demonstrating the essential need to develop clinical strategies that can overcome this barrier, such as the use of specific efflux inhibitors in combination with traditional antifungals.

Advances in molecular phylogeny and fungal taxonomy have reshaped our understanding of *Candida* species, leading to more than just name changes but also promoting the organization of species according to their shared phylogenetic and evolutionary characteristics. These changes reflect taxonomic advances, which, while not directly reflected in the virulence profiles of *Candida* spp., are important for understanding the pathogenic profiles and antifungal sensitivity/resistance across the various clades that comprise the *Candida* genus. The sophisticated virulence factors that favor immune evasion and prolong infections caused by *Candida* spp. highlight the importance of continuous updating in the medical and microbiological fields, revealing how evolutionary knowledge directly impacts the control of fungal infections, improving diagnosis, and tailoring therapies to the profiles of the causative species, aiming to reduce morbidity and mortality associated with fungal infections caused by these species.
